# Pentacyclic Triterpene Bioavailability: An Overview of In Vitro and In Vivo Studies

**DOI:** 10.3390/molecules22030400

**Published:** 2017-03-04

**Authors:** Niege A. J. C. Furtado, Laetitia Pirson, Hélène Edelberg, Lisa M. Miranda, Cristina Loira-Pastoriza, Véronique Preat, Yvan Larondelle, Christelle M. André

**Affiliations:** 1 Departamento de Ciências Farmacêuticas, Faculdade de Ciências Farmacêuticas de Ribeirão Preto, Universidade de São Paulo, Av. do Café, s/n, Ribeirão Preto, São Paulo 14040903, Brazil; 2 Institut des Sciences de la Vie, Université Catholique de Louvain, B-1348 Louvain-la-Neuve, Belgium; pirson.laetitia@gmail.com (L.P.); edelberg@gmail.com (H.E.); mirandamirandalisa@yahoo.fr (L.M.M.); yvan.larondelle@uclouvain.be (Y.L.); 3 Louvain Drug Research Institute, Université Catholique de Louvain, B-1200 Brussels, Belgium; cristina.loira@uclouvain.be (C.L.-P.); veronique.preat@uclouvain.be (V.P.); 4 Department of Environmental Research and Innovation, Luxembourg Institute of Science and Technology, L-4422 Belvaux, Luxembourg

**Keywords:** pentacyclic triterpenes, bioavailability, in vitro studies, in vivo studies

## Abstract

Pentacyclic triterpenes are naturally found in a great variety of fruits, vegetables and medicinal plants and are therefore part of the human diet. The beneficial health effects of edible and medicinal plants have partly been associated with their triterpene content, but the in vivo efficacy in humans depends on many factors, including absorption and metabolism. This review presents an overview of in vitro and in vivo studies that were carried out to determine the bioavailability of pentacyclic triterpenes and highlights the efforts that have been performed to improve the dissolution properties and absorption of these compounds. As plant matrices play a critical role in triterpene bioaccessibility, this review covers literature data on the bioavailability of pentacyclic triterpenes ingested either from foods and medicinal plants or in their free form.

## 1. Introduction

Triterpenes are among the most abundant natural products with approximately 30,000 structures identified to date [1].

From a biological perspective, pentacyclic triterpenes have received much attention, and several of them, including pentacyclic triterpene derivatives, are being marketed as therapeutic agents or dietary supplements around the world [2]. These compounds can be found in several medicinal plants and are natural constituents of the human diet, since they have been found in a great variety of fruits, vegetable oils and cereals [3]. In the Western world, the individual average human consumption of triterpenes is estimated to be approximately 250 mg per day, and in the Mediterranean countries, the average intake could reach 400 mg per day [4].

Beneficial health effects of fruits and vegetables have also been associated with their triterpene content [5]. The number of manuscripts and patents regarding biological activities and the therapeutic potential of triterpenes is increasing as evidenced by searching electronic databases (SciFinder Scholar (6910 references), PubMed (2908 references), Web of Science (2223 references) and Thomson Reuters Integrity (2103 references). Indeed, this class of compounds presents several biological activities, including anti-inflammatory [6], antioxidant [7], anti-viral [8], anti-diabetic [9], anti-tumor [10], hepato-protective [11] and cardio-protective [12] activities.

There is also evidence that pentacyclic triterpenes have the potential to restore vascular disorders associated with hypertension, obesity, diabetes and atherosclerosis [13] and could be used in cancer therapy [14], as anti-ulcer drugs [15], as well as for the prevention and treatment of metabolic diseases [2]. As a result, some triterpenes are currently being evaluated in clinical trials [16,17,18,19].

Although triterpenes showed significant biological activity in in vitro assays [20] and in some animal models [21], the in vivo efficacy in humans is still questioned. Indeed, it depends on many factors, including absorption and metabolism. The oral bioavailability barriers include solubility and/or dissolution, permeation, first-pass metabolism and pre-systemic excretion from the intestine or liver [22].

**Figure 1:**
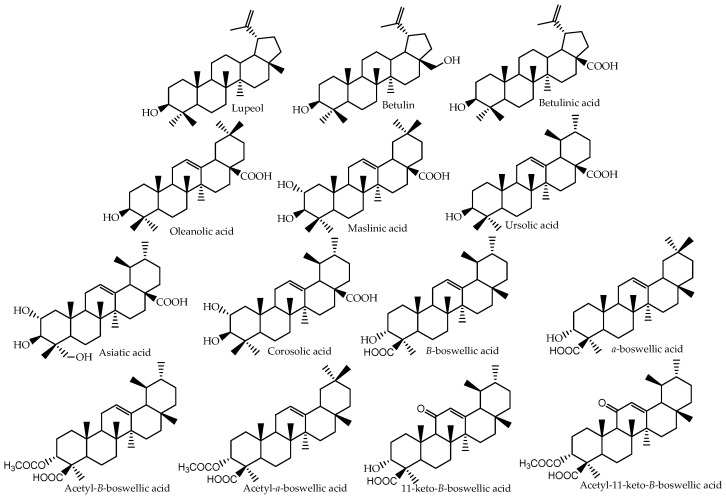
Chemical structures of some bioactive pentacyclic triterpenes.

This review is intended to present an overview of in vitro and in vivo studies that were carried out to determine the bioavailability of pentacyclic triterpenes and to highlight the research that has been performed in order to improve the dissolution properties and absorption of these compounds. The available literature data are presented for the following pentacyclic triterpenes: lupeol, betulin and betulinic acid, which belong to the lupane group; oleanolic, maslinic and alpha-boswellic acids from the oleanane group; and ursolic, asiatic, corosolic and beta-boswellic acids belonging to the ursane group (Figure 1).

## 2. Triterpenes: Chemical Structures and Natural Occurrence

Triterpenes belong to the group of terpenes, which is one of the most widespread groups of natural products. All terpenes are derived from C_5_ isoprene units, and based on the number of isoprene units, terpenes are classified as hemiterpenes (C_5_) monoterpenes (C_10_), sesquiterpenes (C_15_), diterpenes (C_20_), sesterterpenes (C_25_), triterpenes (C_30_) and tetraterpenes (C_40_) [23]. In nature, triterpenoids are often found as tetra- or penta-cyclic structures, but acyclic, mono-, bi-, tri- and hexa-cyclic triterpenes do also exist [2]. The pentacyclic triterpenes can be divided into three main classes: lupane, oleanane and ursane, with each of the classes comprising important bioactive compounds (Figure 1). Triterpenes occur mainly at plant surfaces, such as fruit peel, stem bark or leaves [24]. They are synthesized in the cytosol from the cyclization of an epoxidized squalene that is the precursor of the diverse group of polycyclic triterpenes [25]. These polycyclic structures can occur as free or conjugated triterpenes. Triterpenes may indeed be acylated (with hydroxycinnamic acids for instance [26]) or glycosylated, and in this form, they are called triterpenoid saponins. The name saponin is derived from the Latin word “sapo”, soap. This refers to the tensioactive or surfactant property to produce foam when shaken in aqueous solution, and it happens to be due to the presence of a lipophilic moiety (triterpenoid aglycone, also called sapogenin) bound to a hydrophilic moiety (sugar) [27]. Saponins also cause hemolysis, lysing red blood cells by increasing the permeability of the plasma membrane, and because of this are toxic when injected into the blood stream. However, saponins are relatively harmless when taken orally, and many valuable foods, such as beans, lentils, soybeans, spinach and oat, contain significant amounts of saponins. There are also many examples of medicinal plants containing triterpenoid saponins, such as *Aesculus turbinata* Blume and *Medicago sativa* L., which have been used due to their inhibitory activity on glucose absorption and to their hypocholesterolemic effect, respectively [28]. The sugar moiety of triterpenoid saponins will be digested in the gut by gastrointestinal microorganisms allowing the absorption of the aglycone (triterpene) [28].

Pentacyclic triterpenes have been found in consumed fruits, such as apple peel [29,30], pear peel [24], mango [31], green pepper [31], strawberries [31], mulberry, guava [32] or olives [24,33], but also in aromatic herbs, e.g., basil [32,34], oregano [24], rosemary [35] and lavender [24]. They have also been reported in trees, such as eucalyptus leaves [34] and birch bark [24], as well as in some oriental and traditional medicine herbs widely distributed all over the world [36,37,38]. Besides their low water solubility, they can be found as constituents of decoctions of medicinal plants in which their bioavailability is considered sufficient to promote biological activity [39]. In terms of health effects, boswellic acids have gained a particular interest. They are found as the main active constituents of *Boswellia serrata* Roxb. gum resin extract. This gum resin extract is also known as Indian frankincense and has been used in traditional Eastern medicine for the treatment of inflammatory diseases [18]. This extract is also listed in European pharmacopoeias, and according to Joos et al. [40], among 52% of surveyed German patients with inflammatory bowel disease that use complementary and alternative medicine, 36% have treated inflammatory bowel disease with *B. serrata* extracts.

Mediterranean spices and fruits also contain pentacyclic triterpenes from the lupane, oleanane and ursane groups [41], and maslinic acid, for example, is the main pentacyclic triterpene found in the leaves and fruits of *Olea europaea* L. [42,43,44]. This compound is also gaining acceptance as a potential nutraceutical.

## 3. Bioavailability of Pentacyclic Triterpenes

### 3.1. Definition of Bioavailability and Challenges to Determine Pentacyclic Triterpene Bioavailability in a Complex Matrix

“The term bioavailability is used to indicate the fraction of an orally administered dose that reaches the systemic circulation as intact drug, taking into account both absorption and local metabolic degradation” [45]. In order to measure the absolute oral bioavailability *F*, the plasma drug concentration versus time curves are determined in a group of subjects following oral and intravenous administration. Then, areas under the plasma concentration time curve (AUC) are used to estimate the fraction AUC_oral_/AUC_intravenous_ corrected by the oral and intravenous dose following this formula:

(1) $$F=\frac{AUC_{oral}}{AUC_{intravenous}}\times \frac{Intravenous\;Dose}{Oral\;Dose}\times 100\;\left[\%\right]$$

In the case of pentacyclic triterpenes consumed in medicinal plants and foods, the first step to determine their bioavailability is to evaluate their bioaccessibility, which can be defined as the fraction of ingested nutrients that is released from the food matrix in the gastrointestinal lumen and thereby becomes available for intestinal uptake [46,47].

Foods and medicinal plant matrices have a critical role on the bioavailability of triterpenes because before reaching the small intestine, they undergo digestion within the mouth, stomach and duodenum. They are thus submitted to mechanical actions, enzymatic activities and different pH conditions. In addition, food items are often eaten in conjunction with other foods containing proteins, carbohydrates, fat, fibers and minerals. Proteins, carbohydrates and fibers are able to interact with phytochemical compounds [48,49]; thereby, they might reduce the absorption of lipophilic compounds, such as triterpenes. The presence of fat appears also of major importance, since the solubilization and micellarization of lipophilic compounds are necessary steps prior to absorption [47].

The water insolubility and lipophilicity of pentacyclic triterpenes strongly influence their interactions with components of the absorptive surface within the gastrointestinal tract. The lipophilicity of a compound can be characterized by its partition coefficient between octanol and water (P_ow_), octanol being assumed to have a similar lipophilicity as cell membranes. This coefficient may be used as one of the predictors of drug absorption by passive diffusion [50]. Indeed, intestinal permeability increases with log P_ow_ until values of two, where it reaches a plateau [51]. In contrast, for log P_ow_ of four onwards, the permeability decreases with log P_ow_ because compounds with low aqueous solubility will partition at a slower rate from the cell membrane to the extracellular fluids (transcellular route) [52]. Partition coefficients of pentacyclic triterpenes are reported in Table 1.

Other physicochemical properties of the compounds to be absorbed should also be considered, such as molecular weight, H-bonding with the solvent, intramolecular H-bonding, intermolecular H-bonding, crystallinity, rate of dissolution, polymorphic forms, salt form and ionic charge status [55,56]. In addition, considering that bioactive compounds are consumed in food products and medicinal plants, better knowledge of their physicochemical properties might help to understand their interactions with complex matrices. It is generally accepted that for oral absorption, a molecule should have no more than five hydrogen bond donors and 10 hydrogen bond acceptors, a molecular mass less than 500 and log P not greater than five. As many natural products, triperpenes are an exception to the “rule of five” [57]. Undoubtedly, the activities of digestive enzymes and of the gut microbiota also affect the absorption of bioactive compounds [47]. In general, only aglycones can be absorbed in the small intestine. Prior to absorption, glycosides of pentacyclic triterpenes will thus most probably have to be hydrolysed by intestinal enzymes or by bacterial enzymes in the large intestine.

The degree of absorption of a compound is also related to the surface area over which the absorption is occurring and the time the compound spends in contact with that region [56]. Among the other factors that can influence the degree of absorption of a compound, the pH of the medium from which absorption occurs, the rate of dissolution of the compound and host factors, such as nutrient status, age, genotype, physiological state, infectious disease state or body secretions, are considered as of high importance [56].

The oral bioavailability of a compound also depends on its metabolism, which consists of its biotransformation into other compounds that are usually more water-soluble and more readily excreted in the urine. These biotransformations occur mainly in the liver, but they can also occur in the gastrointestinal tissue, lung, kidney, brain and even blood [56]. They are catalyzed by enzymes that are commonly referred to as drug-metabolizing enzymes, including phase I and phase II metabolizing enzymes. In addition, phase III transporters are responsible for the elimination of the processed or unprocessed compounds from the cells. All together, these proteins provide a barrier against drug penetration and play crucial roles in drug absorption, distribution and excretion [58,59].

Since the determination of pentacyclic triterpene bioavailability in a complex matrix is riddled with obstacles, bioaccessibility is often not taken into account in studies dealing with the bioavailability of pentacyclic triterpenes. Most studies use pure compounds (isolated from medicinal plants, foods or chemically synthesized), precluding the extrapolation of the results obtained to more practical situations where the compounds of interest are consumed in a complex matrix. In addition, in these studies, the terms “absorption” and “bioavailability” are often considered as interchangeable, although absorption represents only one of the steps involved in the passage of a compound from its site of administration into the systemic circulation.

Studies on pentacyclic triterpene bioavailability have been carried out using in vitro assays, animal models and humans. In order to improve the bioavailability of these compounds, different approaches have been performed, including solubility or absorption site affinity increase by technological or chemical modifications of the compounds, the design of micelles, liposomes and nanoparticles [47]. These studies are outlined below.

### 3.2. In Vitro Studies Carried out with Pentacyclic Triterpenes to Predict the In Vivo Bioavailability

In order to reach the bloodstream, released compounds have to cross the intestinal barrier. This can happen by different transport means: passive paracellular diffusion, passive transcellular diffusion, facilitated transport by membrane proteins, active (carrier-mediated) transport and exo-, endo- or trans-cytosis [52].

In vitro models have been developed to study the absorptive processes of compounds administered orally. The reported works indicate good correlation among both in vitro cellular-based and non-cellular-based models and in vivo results [52]. In vitro methods have also been optimized to evaluate the permeability of poorly soluble compounds in order to ensure a high level of accuracy [60].

Lupeol, a lupane-type triterpene, occurs in fruits and vegetables, such as mango, green pepper and strawberries [31]. Although its bioactivities have been well described, only one manuscript [61] reports on a lupeol in vitro permeability study. In this study, permeability experiments were carried out using a Caco-2 cell monolayer grown in a bicameral system. In that kind of experiment, the cell monolayer is allowed to develop on a permeable membrane delimiting two compartments called “apical” and “basolateral”. As these cells get polarized during the differentiation step that follows confluency and since they form tight junctions, the two compartments get physically separated. The apical side represents the intestinal lumen, and the basolateral side represents the systemic circulation. The substance of interest can then be added to the apical compartment, and the system is left to manage the transport of the substance during a determined period of time, often ranging from one to three hours. After the incubation, the medium on each side, as well the cells themselves, is collected and submitted to extraction of the compound of interest for further quantification.

Caco-2 cell monolayers can be used to predict drug transport by different pathways across the intestinal epithelium. The apparent permeability coefficient (*P_app_*), which is a measure of the compound’s ability to cross the intestinal barrier, is calculated using Equation (2), where *Q* is the amount of compounds (µg) transported over time *t* (s), *A* is the surface area of the porous membrane (cm^2^) and *C_D_* is the initial concentration added in the apical side (µg/cm^3^). Substances with a *P_app_* value below 1 × 10^−6^ cm/s, between 1 and 10 × 10^−6^ cm/s and above 10 × 10^−6^ cm/s, are respectively considered as poorly (0%–20%), moderately (20%–70%) or well orally absorbed (70%–100%) in humans [62].

(2) $$P_{app}=\frac{\Delta Q}{\Delta t}\times \frac{1}{A.C_{D}}\left[\mathrm{cm}/s\right]$$

Examining the permeability of lupeol in nanoparticles containing 16% (*w*/*v*) of lupeol, Chairez-Ramírez et al. [61] reported that from the results of transport at 8 h of incubation, only a small fraction of lupeol (traces) was transported from the apical to the basolateral side. However, the authors concluded that although transport across the Caco-2 cell model had not been observed, the best anti-inflammatory effects were observed at the highest dose of pure lupeol (20 µM) and the lowest dose of the nanonutraceutical compound (5 µM), suggesting that this difference could be related to an increased bioavailability of encapsulated lupeol.

**Figure 2:**
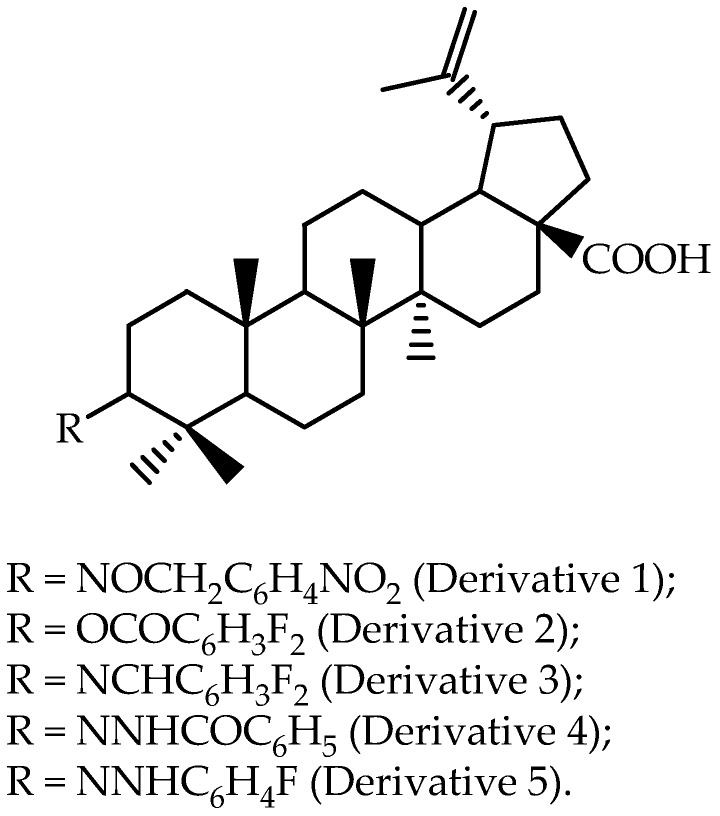
Chemical structures of betulinic acid derivatives evaluated by Rajendran et al. [63].

The pentacyclic triterpene betulinic acid is another lupane-type triterpene that possesses anticancer and anti-HIV activity [8], but besides these effects, its low aqueous solubility results in a low effective concentration and limited absorption in the gastrointestinal tract, seriously limiting its therapeutic applications. Betulinic acid derivatives have thus been synthesized in order to modulate the water solubility of the betulinic acid moiety. These derivatives have been tested with a non-cellular model called the parallel artificial membrane permeability assay (PAMPA™, Millipore, Watertown, MA, USA), in order to rapidly predict the passive transport of betulinic acid derivatives (Figure 2) across a lipid layer that mimics the intestinal lipid bi-layer [63]. The permeability of the betulinic acid derivatives was between 4.9% to 32.7%. Betulinic acid was not detected using this assay considering the limit of quantitation (3 µg/mL). The authors concluded that the betulinic acid derivatives fall in the group of “moderate” to “poor” permeable compounds when compared to known drugs.

Permeability studies were also carried out with oleanolic acid using in vitro Caco-2 cells [64]. Jeong et al. [64] reported that the *P_app_* of oleanolic acid in the apical to basolateral direction at 10 and 20 µM (1.1–1.3 × 10^−6^ cm/s) was similar to that of a low-permeability standard atenolol (0.25 × 10^−6^ cm/s), suggesting that oleanolic acid may be poorly absorbed. In addition, Jeong et al. [64] found that there was no significant difference between the *P_app_* for the apical to basolateral direction and the *P_app_* for the basolateral to apical direction, which also suggests that the transport of oleanolic acid across the intestinal barrier occurs by passive diffusion and is not effluxed by the transporter.

An oral solid dispersion of oleanolic acid prepared by using spray freeze drying technology was evaluated in different formulations in in vitro transport studies using the Caco-2 cell monolayer [65]. The authors reported that the presence of sodium caprate as the wetting agent and permeation enhancer in the formulation increased the permeation of oleanolic acid through the Caco-2 cell monolayer in 2 h by 2.76-times (*p* < 0.05). The increased permeability occurred with a concomitant reduction in the transepithelial electrical resistance (TEER), which is a widely-accepted quantitative technique to measure the integrity of the cell monolayer grown on membrane inserts in cell culture models [66]. Therefore, the authors suggested that this is indicative of an increased transport through the paracellular route via opening of the cellular tight junctions.

**Figure 3:**
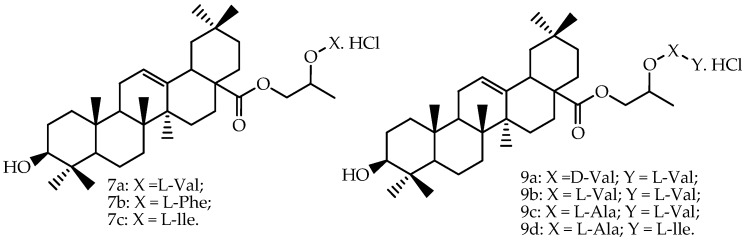
Chemical structures of oleanolic acid prodrugs evaluated by Cao et al. [67].

Prodrugs of oleanolic acid (Figure 3) were also evaluated in Caco-2 permeability experiments, and all prodrugs showed the following increases of permeability through the Caco-2 cell monolayer compared to oleanolic acid: **7a** (5.27-fold), **9b** (3.31-fold), **9a** (2.26-fold), **7b** (2.10-fold), **7c** (2.03-fold), **9c** (1.87-fold) and **9d** (1.39-fold) [66]. The authors mentioned that the transepithelial electrical resistance (TEER) was little changed, indicating that the tight junctions were not opened.

One permeability study was performed in vitro with ursolic acid, either as free compound and in an ethanol extract from *Salvia officinalis* L. That study used human intestinal epithelial Caco-2 cell monolayers [68]. The content in ursolic acid of the ethanol extract from *S. officinalis* was determined by HPLC analysis as 2.6 ± 0.4 g/L. Ursolic acid and the *S. officinalis* extract at 2, 5, 10 and 20 µM (non-cytotoxic concentrations) were added to the apical chamber of the bicameral system, and then, basolateral solutions were collected after 0.5, 1, 2 and 4 h. After 4 h, the authors also analyzed the apical and cellular compartments. The uptake of ursolic acid as free compound and ursolic acid in *S. officinalis* extract increased linearly and significantly from 0.03 ± 0.01–0.2 ± 0.04 µM/h/cm^2^ and was not saturable across the evaluated concentrations (5–20 µM) and the tested time points (0.5–4 h), suggesting an uptake by passive diffusion. No significant differences were found between ursolic acid as free compound or ursolic acid in the plant extract since the permeability coefficients were of 2.8 ± 0.1 × 10^−6^ cm/s for ursolic acid free compound and 2.5 ± 0.4 × 10^−6^ cm/s for ursolic acid in plant extract.

Yuan et al. [69] reported an investigation of asiatic acid absorption using Caco-2 cell line in vitro model*.* After transportation of 2 µM of asiatic acid across the Caco-2 cell monolayer from the apical to basolateral and from basolateral to apical side, the permeabilities of asiatic acid were determined to be more than 1 × 10^−5^ cm/s, which indicates a good absorption. The results obtained with a rat intestinal perfusion model also showed that the permeabilities were more than 1 × 10^−5^ cm·s^−1^, confirming the absorption results in the Caco-2 cell absorption model.

A significant number of studies reported investigations of the absorption of boswellic acids in human Caco-2 cell lines. Krüger et al. [70] examined the permeability of 11-keto-β-boswellic acid and 3-acetyl-11-keto-β-boswellic acid, as well as their interaction with three transporters: the organic anion transporter polypeptides family member 1B3 (OATP1B3), the multidrug resistance-associated protein 2 (MRP2) and P-glycoprotein. They also evaluated a *B. serrata* extract. The experiments were carried out using 10 µM of 11-keto-β-boswellic acid and 9.5 µM of 3-acetyl-11-keto-β-boswellic acid as free compounds and in the extract. Considering the apparent permeability coefficient obtained for the isolated 11-keto-β-boswellic acid (*P_app_* = 1.69 × 10^−6^ cm/s), this compound can be classified as moderately absorbed. The *P_app_* value determined for this compound in the crude extract was 2.14 × 10^−6^ cm/s. The isolated 3-acetyl-11-keto-β-boswellic acid was not detected when permeability studies were carried out using the isolated compound and the complex extract. The authors also reported that at the end of absorptive transport experiments, a significant loss of mass balance was detected for both compounds, but as they carried out control experiments in the absence of the Caco-2 monolayer, they concluded that these compounds were mainly accumulated in and/or adsorbed on the cell monolayer. Both compounds modulated the activity of OATP1B3 and MRP2 transporters, indicating that therapeutic relevant interactions with other anionic drugs may be expected. The authors also concluded that both compounds are not substrates of P-glycoprotein.

Permeability studies on Caco-2 cell lines were also carried out with 11-keto-β-boswellic acid and 3-acetyl-11-keto-β-boswellic acid by Hüsh et al. [71]. Interestingly, these authors developed formulations that increased the solubility of boswellic acids up to 54-times. One of the formulations (extract-phospholipid complex) increased the mass flux of 11-keto-β-boswellic and 3-acetyl-11-keto-β-boswellic acids by eight- and 15-times, respectively, in comparison with a non-formulated extract.

Other boswellic acids were evaluated in Caco-2 permeability studies by Gerbeth et al. [72], and the obtained apparent permeability coefficients are presented in Table 2. These authors reported that the permeabilities of 11-keto-β-boswellic and 3-acetyl-11-keto-β-boswellic acids were underestimated in previous experiments and that their adapted Caco-2 model, which was closer to physiological conditions thanks to the addition of bovine serum albumin to the basolateral side and the use of modified fasted state simulated intestinal fluid on the apical side, provided better prediction of the absorption in vivo. Considering the classification of compounds in poorly, moderately and well absorbable based on the apparent permeability coefficient values reported by Yee et al. [62], 11-keto-β-boswellic and 3-acetyl-11-keto-β-boswellic acids can be considered as well absorbable (70%–100%) according to the results obtained by Gerbeth et al. [72].

### 3.3. Bioavailability of Bioactive Pentacyclic Triterpenes In Vivo

Betulinic acid bioavailability has been reported in in vivo studies by Udeani et al. [73] and Godugu et al. [74]. Udeani et al. [73] performed a pharmacokinetic study on betulinic acid and showed that it is widely distributed in several tissues after a 500-mg/kg intraperitoneal administration to mice. In this study, serum samples were obtained after a 250 or 500 mg/kg intraperitoneal dose of betulinic acid. A two-compartment, first order model was selected for pharmacokinetic modeling. Godugu et al. [74] reported an improvement of pharmacokinetic parameters of betulinic acid when this compound was evaluated in an optimized formulation in spray-dried mucoadhesive microparticles. These authors reported a significant increase in the oral bioavailability of betulinic acid (Table 3) that resulted in a superior anticancer effect when evaluated in mice A549 orthotopic lung cancer models and in metastatic tumor models. The lung tumor weights and volumes were significantly reduced upon oral administration of this formulation at the dose of 100 mg/kg, daily for three weeks.

Other studies have reported on different formulation approaches of betulinic acid, such as complexation with gamma-cyclodextrin [75], beta-cyclodextrin [76] and nanoemulsion [77], but in these manuscripts, the authors reported only an improvement of the anticancer effect evaluated in in vitro tests on tumor cell lines and in vivo tests on experimental animal models.

**Figure 4:**
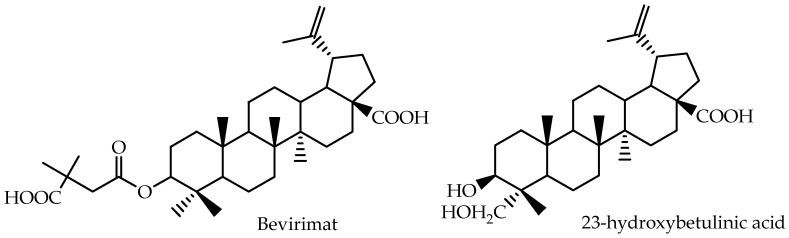
Chemical structures of betulinic acid derivatives evaluated by Yang et al. [78] and Martin et al. [79].

The pharmacokinetics of different betulinic acid derivatives have also been studied [78,79,80] and the chemical structures of these derivatives are presented in Figure 4.

Rajendran et al. [63] reported that, based on in vitro results, a dihydro-betulinic acid derivative modified at the C-3 position (4-nitrobenzyl-oximino), as presented as Derivative 1 in Figure 2, was selected to perform an in vivo assay using male Wistar rats. Non-compartmental analysis was selected for pharmacokinetic modeling, and the reported data are presented in Table 3. The authors found that this Derivative 1 showed favorable pharmacokinetic characteristics and better in vivo antitumor efficacy as compared to betulinic acid in a human colon cancer xenograft model.

Yang et al. [78] developed a new assay based on liquid chromatography/mass spectrometry for the quantitative analysis of 23-hydroxybetulinic acid in mouse plasma after intragastric administration. A two-compartment, first order model was selected for pharmacokinetic modeling, and the reported data are also presented in Table 3. 23-hydroxybetulinic acid was found to have a long elimination half-live of 25.6 h and low bioavailability of 2.3%.

Bevirimat [3-O-(3′,3′-dimethylsuccinyl)-betulinic acid], a betulinic acid derivative, represents a promising class of anti-HIV agents with a novel mechanism. It inhibits HIV-1 maturation by blocking the cleavage of p25 to functional p24, resulting in the production of noninfectious HIV-1 particles [80]. Martin et al. [79] calculated AUC_p.o_ for different bevirimat oral doses and showed that the compound presents a dose-proportional pharmacokinetics during repeated dosing for 10 days. The pharmacokinetic parameters of bevirimat were estimated using non-compartmental methods, and the reported data for Day 10 are presented in Table 3.

According to the literature, in mouse, rat or dog plasma, betulinic acid was reported to be 99.99% bound to serum proteins [81].

**Figure 5:**
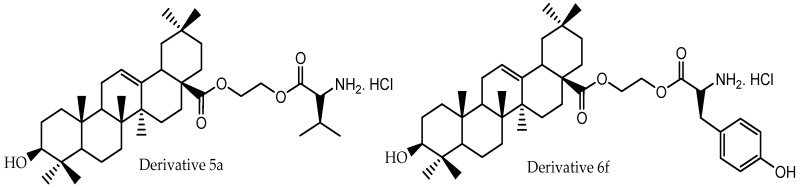
Chemical structures of oleanolic acid derivatives evaluated by Cao et al. [82].

Regarding oleanolic acid, pharmacokinetic parameters of this triterpene and of two amino acid ester prodrugs of oleanolic acid (**5a** and **6f**, Figure 5) were reported by Cao et al. [82] after oral administration to rats that received by oral gavage 300 mg/kg of oleanolic acid and its prodrugs (Table 4). Standard non-compartmental analysis was performed for the estimation of the absorption profile using Kinetica^®^, Version 4.4 (Thermo Electron Corporation, New York, NY, USA). The authors concluded that the water solubility of the prodrugs of oleanolic acid was greater than that of oleanolic acid. The permeability studies with rats in a single-pass intestinal perfusion model showed that all of the prodrugs had higher membrane effective permeability, and **5a** and **6f** exhibited enhanced oral bioavailability of oleanolic acid in rats.

In another work [67], the same group of researchers also determined the pharmacokinetic parameters of two amino acid/dipeptide diester prodrugs with a propylene glycol link to oleanolic acid (**7a** and **9b**), and the reported data are presented in Table 4. Compared to the ethylene glycol-linked amino acid/dipeptide diester prodrugs of oleanolic acid synthesized by Cao et al. [82], the results from this study revealed that part of the propylene glycol-linked amino acid/dipeptide diester prodrugs showed better stability, permeability, affinity and bioavailability. The chemical structures of these derivatives are presented in Figure 3. Standard non-compartmental analysis was performed for the estimation of the absorption profile using Kinetica^®^, Version 4.4.

Jeong et al. [64] reported on the oleanolic acid pharmacokinetic parameters in rats after intravenous injection at doses of 0.5, 1 and 2 mg/kg and oral administration at doses of 10, 25 and 50 mg/kg (Table 4). According to these authors, oleanolic acid was also metabolically unstable following incubation with rat liver microsomes in the presence of NADPH. These authors concluded that the low bioavailability of 0.7% of oleanolic acid determined after oral administration to rats may be due to a poor gastrointestinal absorption and subsequent hepatic microsomal metabolism.

Pharmacokinetic parameters of some formulations of oleanolic acid were determined by Tong et al. [65] after 50 mg/kg oral administration to rats. The authors evaluated three different formulations: Formula B (solid dispersion of oleanolic acid in polyvinylpyrrolidone-40 matrix using spray freeze drying), Formula F (solid dispersion of oleanolic acid in polyvinylpyrrolidone-40 matrix using spray freeze drying and the addition of sodium caprate) and Formula G (solid dispersion of oleanolic acid sodium salt in polyvinylpyrrolidone-40 matrix using spray freeze drying and the addition of sodium caprate). The reported data are presented in Table 4. Comparison of Formulas B and G revealed that the addition of sodium caprate resulted in an initial boost of oleanolic acid concentration in the plasma, reaching a peak within the first 13–18 min and dropping progressively. The influence of the sodium salt form on the oral bioavailability of oleanolic acid was assessed by comparing the results that were achieved with Formulas F and G, and the authors concluded that the replacement of oleanolic acid with its sodium salt did not exert significant impact on either the plasma concentration-time profile or the associated kinetic parameter estimates.

Xi et al. [83] developed a self-nanoemulsified drug delivery system of oleanolic acid and evaluated in vivo oral bioavailability in rats and compared this formulation to the commercially-available oleanolic acid tablet. The pharmacokinetic parameters are presented in Table 4. According to the authors, the self-nanoemulsified drug delivery system of oleanolic acid showed a 2.4-fold increase in the oral bioavailability of oleanolic acid and an increased mean retention time of oleanolic acid in rat plasma.

Jiang et al. [84] reported dual strategies to improve oral bioavailability of oleanolic acid. They carried out a pharmacokinetic study with a solidified phospholipid complex (oleanolic acid phospholipid complex (OPCH)) composed of oleanolic acid phospholipid complex and hydroxyapatite and the same complex added with ketoconazole (KCZ), since this compound is a noncompetitive inhibitor of CYP3A enzymes. The study was performed in rats after oral administration of 50 mg/kg of oleanolic acid, oleanolic acid phospholipid complex and oleanolic acid phospholipid complex added with ketoconazole. The reported pharmacokinetic parameters are presented in Table 4. The formulation of solidified phospholipid complex and co-administration of ketoconazole improved the bioavailability of oleanolic acid by increasing the solubility and permeability in combination with inhibiting the metabolism of oleanolic acid.

Yang et al. [85] developed a self-microemulsifying drug delivery system to enhance the solubility and bioavailability of oleanolic acid, and a pharmacokinetic study was carried out in rats to compare the developed formulation with the conventional tablet. The reported pharmacokinetic parameters are presented in Table 4. The self-microemulsifying drug delivery system increased the bioavailability of oleanolic acid to 507% by keeping the drug in a dissolved form that contributed to enhance the absorption. In addition, the developed drug delivery system forms a fine oil/water microemulsion with a droplet size of less than 100 nm that provides a large interfacial surface area for the drug. The authors also reported that the high surfactant content in the developed formulation may increase the permeability by disturbing the cell membrane.

A nanosuspension of oleanolic acid stabilized with sucrose ester was developed by Li et al. [86] who carried out pharmacokinetic studies in rats following intravenous (2 mg/kg) and oral administration (10 and 20 mg/kg). The authors compared the developed formulation with an oleanolic acid coarse suspension. The reported non-compartmental model pharmacokinetic parameters are presented in Table 4. The oral bioavailability of the oleanolic acid nanosuspension was 6–7-times higher than that of the oleanolic acid coarse suspension.

Oleanolic acid can also be found in grape skins and raisins (*Vitis vinifera* L.). According to Kanellos et al. [89], the level of oleanolic acid in human plasma reached its highest concentration (24.4 ± 14.4 ng/mL) 4 h post-consumption of 144 g of raisins.

Pharmacokinetic studies of oleanolic acid were carried out in beagle dog by Shi et al. [87] and Li et al. [88]. Shi et al. [87] determined pharmacokinetic parameters of oleanolic acid after oral and intravenous administration of calenduloside E, a triterpene saponin of oleanolic acid conjugated with glucuronic acid, while Li et al. [88] determined pharmacokinetic parameters of a solid dispersion of oleanolic acid prepared with fumed silica by a supercritical fluid technology (Table 4). According to Shi et al. [87], oleanolic acid was found in the plasma after administration of oral doses of calenduloside E, indicating its formation from its glucuronic acid conjugate. In fact, after oleanolic acid formation in the gut, this compound is absorbed and then transported to the liver where it is converted back to its glucuronic acid conjugate. This conjugate undergoes biliary excretion and is transported through the bile to the gut where it is again hydrolyzed to oleanolic acid, and the cycle is repeated. On the basis of the AUC values (Table 4), Li et al. [88] concluded that the solid dispersion of oleanolic acid prepared with fumed silica bioavailability was 1.9-fold higher, as compared with commercial tablets.

Concerning maslinic acid, the main pentacyclic triterpene found in the leaves and fruits of *Olea europaea* L. [43], pharmacokinetic parameters of this triterpene were determined after intravenous (1 mg/kg) and oral (50 mg/kg) administration to rats [90]. Plasma concentrations of maslinic acid versus time were analyzed following a non-compartmental approach by population-based compartmental modeling with the nonlinear mixed-effects approach. Estimates were confirmed by non-compartmental calculations. Some of the pharmacokinetic parameters estimated through the noncompartmental and compartmental approach were: AUC_0→∞_ µmol·h/L = 5.06 and AUC_0→∞_ µmol·h/L = 5.17 after intravenous injection; AUC_0→∞_ µmol·h/L = 14.87 and AUC_0→∞_ µmol·h/L = 12.43 after oral administration; C_0_ = 32.79 µM and C_0_ = 17.61 µM after intravenous injection; C_max_ = 5.36 µM and C_max_ = 4.03 µM after oral administration. The oral bioavailability of maslinic acid was determined to be 5.13%. This low bioavailability could be due to either a first-pass effect of the compound at the gut wall or the liver or a poor gastrointestinal absorption.

Regarding ursolic acid, concentrations in mice tissues and plasma were determined after intravenous administration of 15 mg/kg of ursolic acid dissolved in a 10-mL mixture of ethanol and polyethylene glycol 400 (1:1) and 15 mg/kg of ursolic acid phospholipid nanoparticles [91]. The plasma concentration of ursolic acid reached after 12 h of intravenous administration in phospholipid nanoparticles (2.07 ng/mL) was higher than the plasma concentration reached with the ursolic acid solution (0.82 ng/mL).

Other formulation approaches have been developed for improving the dissolution properties and bioavailability of ursolic acid. Nanoparticles were prepared using different procedures by Zhi-Qiang et al. [92] and Yang et al. [93], and liposomes were prepared by Yang et al. [94]. Pharmacokinetic studies were carried out by these authors in rats or mice, and the reported results are presented in Table 5. It should be highlighted from the results achieved by Zhi-Qiang et al. [92] that the oral bioavailability of ursolic acid nanoparticles prepared using d-α-tocopheryl polyethylene glycol 1000 succinate was 27.5-fold higher than that of the ursolic acid free compound.

Asiatic acid, an ursane type triterpene, is the bioactive constituent of *Centella asiatica* (L.) Urb. extract that is marketed by Syntex in a number of European Union countries and Canada under the trade name Madecassol^®^ to treat various dermatological conditions, including burns [95]. Asiatic acid is found in *C. asiatica* as free triterpene and as asiaticoside (triterpenoid saponin in which the aglycone is the triterpene asiatic acid). The bioavailability of asiatic acid was studied in 12 healthy male and female volunteers after oral administration of equimolar doses of asiatic acid (12 mg) and asiaticoside (24 mg). Pharmacokinetic parameters were determined, and the difference in the mean AUC between treatments was less than 2% (AUC_0→12 h_ ng·h/mL = 614 ± 250 after asiatic acid administration on Day 10 of a twice daily regime and AUC_0→12 h_ ng·h/mL = 606 ± 316 after asiaticoside administration on Day 10 of a twice daily regime). However, the C_max_ reached after asiatic acid administration was higher (C_max_ = 97.8 ± 43.5 ng/mL) than the C_max_ reached after asiaticoside administration (C_max_ = 65.1 ± 30.4 ng/mL), and T_max_ was slightly shorter on asiatic acid (T_max_ = 4.0 ± 2.5 h) than asiaticoside (T_max_ = 5.4 ± 4.3 h). Asiaticoside thus contributes to the plasma levels of asiatic acid after Madecassol^®^ administration though in vivo hydrolysis of asiaticoside into asiatic acid. According to the authors, the combination of asiatic acid and asiaticoside in Madecassol^®^ provides both a rapid and a prolonged availability of asiatic acid for maintained therapeutic effectiveness during the dose interval.

Yuan et al. [69] reported the determination of plasma concentrations of asiatic acid after oral (20 mg/kg) and intravenous (2 mg/kg) administration, and the pharmacokinetic parameters were calculated by using DAS Version 3.0 according to the non-compartmental model. According to the authors, asiatic acid can be absorbed into blood rapidly since the maximum plasma concentration was reached at 30 min (C_max_ = 0.394 ng/mL). However, the small *t*_1/2_ = 0.348 h suggested that asiatic acid may be metabolized quickly by hepatic enzymes, and the authors confirmed this hypothesis through investigations on the metabolic rate of asiatic acid in rat liver microsomes. The absolute oral bioavailability of asiatic acid was determined to be 16.25%.

Lingling et al. [96] developed solid lipid nanoparticles of asiatic acid tromethamine salt to enhance the oral bioavailability and performed a pharmacokinetic study in rats. The main pharmacokinetic parameters were determined for solid lipid nanoparticles of asiatic acid tromethamine salt: AUC_0→∞_ µg·h/L = 2347.1 ± 238.4; C_max_ = 680.0 ± 233.5 µg/L and T_max_ = 0.25 ± 0.0 h. The pharmacokinetic parameters were also determined for asiatic acid tromethamine salt: AUC_0→∞_ µg·h/L = 929.9 ± 238.4; C_max_ = 184.0 ± 70.8 µg/L and T_max_ = 0.25 ± 0.0 h. 

Corosolic acid, another ursane-type triterpene, is one of the bioactive triterpenes found in *Potentilla discolor* Bunge, which has been used for diabetes in China for a long time [97]. Li et al. [97] studied the pharmacokinetics of corosolic acid after oral administration of the *P. discolor* extract (1.33 g/kg) to normal and diabetic rats. Pharmacokinetic parameters of corosolic acid revealed significant differences between normal and diabetic rats. The AUC_0→∞_ and C_max_ were elevated in diabetic rats (AUC_0→∞_ mg·h/L = 3.26 ± 0.28 and C_max_ = 0.49 ± 0.03 mg/L) and compared with the values obtained for normal rats (AUC_0→∞_ mg·h/L = 1.42 ± 0.04 and C_max_ = 0.31 ± 0.07 mg/L). The results showed a double-peak profile in the corosolic acid plasma concentration after around 0.5 and 2 h, with peak concentrations around 0.25 and 0.27 µg/mL, respectively. These data are different from those obtained in a study reported by Liu et al. [98], in which a maximum plasma concentration of 0.30 µg/mL within 3 h was reached at 9.2 min and presented a single-peak profile after oral administration of corosolic acid (20 mg/kg). According to Li et al. [97], these differences might be due to the fact that in the study carried out by Liu et al. [98], corosolic acid was provided as pure compound (20 mg/kg), while in the study of Li et al. [97], corosolic acid was administered in the form of herbal extract (45.3 mg/kg of corosolic acid in 1.33 g/kg of herbal extract). The AUC_0→∞_ of corosolic acid in normal rats was only 1.42 mg·h/L, suggesting that only traces of corosolic acid can be absorbed into the blood when the compound is orally administered in an herbal extract.

Boswellic acids, which are constituents of *B. serrata* gum resin extract, are some of the most studied pentacyclic triterpenes, and several clinical studies have confirmed their anti-inflammatory and antitumor activities [99]. Pharmacokinetic studies have been carried out in humans, and the reported data are presented in Table 6.

Sharma et al. [19] carried out pharmacokinetic study of 11-keto-β-boswellic acid in twelve healthy male volunteers between 18 and 50 years of age after oral single dose of Wok Vel™ capsule containing the standardized *B. serrata* gum extract with a minimum of 65% organic acids or minimum 40% total boswellic acids. Blood samples were withdrawn prior to drug administration and at 30, 60, 120, 150, 180, 210, 240, 300, 360, 480, 600, 720 and 840 min after drug administration. The authors performed a noncompartmental pharmacokinetic analysis of concentration time data. A single dose administration of 333 mg of the standardized *B. serrata* did not cause side effects. Considering the elimination half-life, the standardized *B. serrata* extract needs to be given orally at the interval of six hours, and the steady state is reached after approximately 30 h.

Sterk et al. [18] reported that the pharmacokinetic profile of boswellic acids after oral administration of an extract is influenced by food intake. They determined the pharmacokinetic parameters after oral administration of 786 mg of *B. serrata* dry extract (55.08% of boswellic acids) in twelve healthy male volunteers. This study was also a single dose study, but was under normal and high-fat meal. Blood samples were collected at 0.5, 1, 2, 3, 4, 8, 12, 18, 24, 36, 48 and 60 h. The pharmacokinetic profile of boswellic acid varied with food intake, and a better absorption of boswellic acids occurred in the high fat-meal due to the presence of bile acids.

Skarke et al. [17] also determined the pharmacokinetic parameters of 11-keto-β-boswellic and 3-acetyl-11-keto-β-boswellic acids in twelve male volunteers after oral administration of a single oral dose of two Boswelan capsules containing 800 mg of *Boswellia serrata* extract. Blood samples were collected at 1, 2, 3, 4, 6, 8, 12 and 24 h after oral dosing. In this study, food intake also affected the bioavailability of boswellic acids, but caused less effect than those caused in the study reported by Sterk et al. [18].

Novel approaches have been developed to enhance the bioavailability and consequently the bioactivity of boswellic acids in humans, including the synthesis of new derivatives [100,101] and the preparation of different formulations composed of *B. serrata* extracts [102,103] or isolated boswellic acids [104,105]. Therefore, all of these approaches may contribute to providing new therapeutic candidates for inflammation, cancer and other diseases.

## 4. Conclusions

Studies on pentacyclic triterpenes’ bioavailability showed that a wide number of factors can influence the oral bioavailability of these compounds. Interestingly enough, triterpene bioavailability can be improved by increasing the poor solubility in the gastrointestinal fluid and their absorption, as well as, in some situations, by inhibiting their metabolism. The bioavailability of pentacyclic triterpenes determined in in vivo studies has been show to differ when these compounds are administered as pure compounds or in a complex matrix, such as a food item. More specifically, high fat meals enhance triterpene absorption. Different strategies developed to improve the oral bioavailability of pentacyclic triterpenes reported herein demonstrate that dietary supplements and therapeutic agents may be developed with these compounds to provide health benefits and to treat diseases.

## Figures and Tables

**Table 1:** Octanol/water partition coefficients (P_ow_; expressed as log P_ow_) of pentacyclic triterpenes.

Compound	log P_ow_	Reference
Lupeol	7.45	Predicted by ChemAxon software
Betulin	6.17	Predicted by ChemAxon software
Betulinic acid	6.73	[53]
Oleanolic acid	6.47	[53]
Maslinic acid	5.52	Predicted by ChemAxon software
Ursolic acid	6.43	[53]
Asiatic acid	5.80	[54]
Corosolic acid	5.51	Predicted by ChemAxon software
β-boswellic acid	6.58	Predicted by ChemAxon software

**Table 2:** Mean of apparent permeability coefficient values reported for boswellic acids by Gerbeth et al. [72].

Compound	*P_app_* Value × 10^−6^ cm/s
11-keto-β-boswellic acid	29.54
3-acetyl-11-keto-β-boswellic acid	17.83
β-boswellic acid	4.47
3-acetyl-β-boswellic acid	6.18
α-boswellic acid	5.52
3-acetyl-α-boswellic acid	4.72

**Table 3:** Pharmacokinetic parameters of betulinic acid in different formulations and of betulinic acid derivatives.

Compound	Species (Sample)	Dose (mg/kg)	Route of Administration	AUC_0→∞_ (µg·h/mL)	C_max_ (µg/mL)	T_max_ (h)	Reference
Betulinic acid	Mice (serum)	500	intraperitoneal	39.9	4.00	0.22	[73]
Betulinic acid	Mice (serum)	250	intraperitoneal	18.4	2.21	0.15	[73]
Betulinic acid	Mice (skin)	500	intraperitoneal	3504.0	300.9	3.90	[73]
Betulinic acid	Rat (plasma)	100	oral	7.26 ± 1.65	1.16 ± 0.22	2.36 ± 0.38	[74]
BA-SD	Rat (plasma)	100	oral	53.86 ± 7.79	4.54 ± 0.25	3.17 ± 0.85	[74]
23-Hydroxybetulinic acid	Mouse (plasma)	200	intragastric	24.9	3.1	2	[78]
Bevirimat	Human (plasma)	200	oral	1113.7 ± 216.7	58.0 ± 10.83	1.50 (0.8–3.0)	[79]
Derivative 1	Rat (plasma)	10	intravenous	43.6 ± 6.3	101.5 ± 21.7	0.05 ± 0.0	[63]

BA-SD: betulinic acid in spray-dried mucoadhesive microparticles; bevirimat: 3-*O*-(3′,3′-dimethylsuccinyl)-betulinic acid; Derivative 1: the chemical structure is presented in Figure 2; (AUC_0→∞_): area under the plasma concentration-time curve from zero to infinity time; C_max_: maximum plasma concentration; T_max_: time to maximum concentration. Values are expressed as the mean ± standard error of the mean when available.

**Table 4:** Pharmacokinetic parameters of oleanolic acid in different formulations and of oleanolic acid derivatives.

Compound	Specie (Sample)	Dose (mg/kg)	Route of Administration	AUC_0→∞_ (µg·min/mL)	C_max_ (µg/mL)	T_max_ (h)	Reference
Oleanolic acid	rat (plasma)	0.5	intravenous	16.0 ± 1.9	N.A.	N.A.	[64]
Oleanolic acid	rat (plasma)	1	intravenous	32.6 ± 10.4	N.A.	N.A.	[64]
Oleanolic acid	rat (plasma)	2	intravenous	71.6 ± 12.7	N.A.	N.A.	[64]
Oleanolic acid	rat (plasma)	10	oral	N.A.	N.A.	N.A.	[64]
Oleanolic acid	rat (plasma)	25	oral	5.9 ± 5.5	0.074 ± 0.06	0.42 ± 0.30	[64]
Oleanolic acid	rat (plasma)	50	oral	10.7 ± 10.0	0.132 ± 0.12	0.35 ± 0.28	[64]
Oleanolic acid	rat (plasma)	300	intragastric	N.A.; AUC_0→24_ (µg·h/mL) = 4.98 ± 0.42	0.47 ± 0.034	0.50	[82]
Oleanolic acid prodrug **5a**	rat (plasma)	300	intragastric	N.A.; AUC_0→24_ (µg·h/mL) = 10.99 ± 0.65	0.73 ± 0.067	0.83 ± 0.22	[82]
Oleanolic acid prodrug **6f**	rat (plasma)	300	intragastric	N.A.; AUC_0→24_ (µg·h/mL) = 10.14 ± 1.14	0.72 ± 0.070	0.58 ± 0.17	[82]
Oleanolic acid prodrug **7a**	rat (plasma)	300	intragastric	N.A.; AUC_0→24_ (µg·h/mL) = 17.68 ± 3.07	1.43 ± 0.17	1.25 ± 1.37	[67]
Oleanolic acid prodrug **9b**	rat (plasma)	300	intragastric	N.A.; AUC_0→24_ (µg·h/mL) = 16.88 ± 2.84	1.23 ± 0.24	1.67 ± 1.81	[67]
Formula B			oral	N.A.; AUC_0→t_ (ng·min/mL) = 40,216.98 ± 31,860.38	0.16 ± 0.11	0.80 ± 0.45	[65]
Formula F			oral	N.A.; AUC_0→t_ (ng·min/mL) = 31,067.44 ± 17,840.92	0.39 ± 0.18	0.21 ± 0.16	[65]
Formula G			oral	N.A.; AUC_0→t_ (ng·min/mL) = 32,657.41 ± 11,832.92	0.34 ± 0.16	0.26 ± 0.15	[65]
Commercial oleanolic acid tablet	rat (plasma)	50	oral	N.A.; AUC_0→t_ (ng·min/mL) = 14,974.89 ± 10,906.19	0.10 ± 0.06	0.80 ± 0.45	[83]
OA SEDDS	rat (plasma)	50	oral	N.A.; AUC_0→t_ (ng·min/mL) = 36,041.38 ± 28,965.03	0.09 ± 0.04	1.5 ± 1.21	[83]
Oleanolic acid	rat (plasma)	50	oral	N.A.; AUC_0→t_ (ng·min/mL) = 15,576 ± 1378.8	0.059 ± 0.01	0.313 ± 0.12	[84]
OPCH	rat (plasma)	50	oral	N.A.; AUC_0→t_ (ng·min/mL) = 21,636 ± 1147.8	0.078 ± 0.01	0.46 ± 0.001	[84]
OPCH with KCZ	rat (plasma)	50	oral	N.A.; AUC_0→t_ (ng·min/mL) = 42,462 ± 1812.6	0.131 ± 0.01	0.25 ± 0.00	[84]
SMEDDS	rat (plasma)	50	oral	106.51 ± 9.47	0.209 ± 0.04	2.00 ± 1.00	[85]
Oleanolic acid tablet	rat (plasma)	50	oral	21.00 ± 4.42	0.077 ± 0.01	2.75 ± 0.50	[85]
OANS	rat (plasma)	2	intravenous	121.49 ± 27.37	21.98 ± 5.79	N.A.	[86]
OANS	rat (plasma)	10	oral	21.35 ± 3.89	0.39 ± 0.17	0.21 ± 0.07	[86]
OANS	rat (plasma)	20	oral	44.06 ± 7.25	0.81 ± 0.25	0.35 ± 0.13	[86]
OA coarse suspension	rat (plasma)	20	oral	6.74 ± 3.42	0.06 ± 0.04	0.21 ± 0.07	[86]
Calenduloside E	Beagle dogs (plasma)	4.2	oral	N.A.; AUC_0→t_ (ng·h/mL) = 83.51 ± 26.91	0.013 ± 0.004	1.33 ± 0.52	[87]
Calenduloside E	Beagle dogs (plasma)	2.1	intravenous	N.A.; AUC_0→t_ (ng·h/mL) = 395.19 ± 167.79	1.057 ± 0.591	0.083 ± 0.00	[87]
Commercial oleanolic acid tablet	Beagle dogs (plasma)	6.6	oral	N.A.; AUC_0→24_ (ng·h/mL) = 128.87 ± 37.55	0.03 ± 0.005	1.50 ± 0.45	[88]
OA-silica capsules	Beagle dogs (plasma)	6.6	oral	N.A.; AUC_0→24_ (ng·h/mL) = 228.51 ± 20.35	0.07 ± 0.01	1.17 ± 0.26	[88]

OA SEDDS: a self-nanoemulsified drug delivery system of oleanolic acid; OPCH: oleanolic acid phospholipid complex; OPCH with KCZ: oleanolic acid phospholipid complex with ketoconazole; SMEDDS: self-microemulsifying drug delivery system loaded with oleanolic acid; OANS: oleanolic acid nanosuspension; OA coarse suspension: oleanolic acid coarse suspension; calenduloside E: 3-*O*-[β-d-glucuronopyranosyl]oleanolic acid; OA-silica capsules: optimized solid dispersion of oleanolic acid in capsule; (AUC_0→∞_): area under the plasma concentration-time curve from zero to infinity time; (AUC_0→t_): area under the concentration time-curve; AUC _0→24_: area under the curve between 0 and 24 h; C_max_: maximum plasma concentration; T_max_: time to maximum concentration. Values are expressed as the mean ± standard error of the mean when available. N.A.: non-available data.

**Table 5:** Pharmacokinetic parameters of ursolic acid in different formulations.

Compound	Specie (Sample)	D (mg/kg)	Route of Administration	AUC_0→12_ (µg·h/mL)	C_max_ (µg/mL)	T_max_ (h)	Reference
Ursolic acid	rat (plasma)	10	oral	1.37 ± 0.43	1.17 ± 0.27	0.75 ± 0.07	[92]
Ursolic acid nanoparticles freshly prepared	rat (plasma)	10	oral	36.57 ± 1.90	9.32 ± 0.46	0.5 ± 0.04	[92]
Ursolic acid	rat (plasma)	100	oral	0.98 ± 0.05	0.29 ± 0.27	1.2 ± 0.3	[93]
Ursolic acid nanoparticles	rat (plasma)	100	oral	2.84 ± 0.11	1.27 ± 0.12	1.1 ± 0.2	[93]
Ursolic acid	mice (plasma)	20	intravenous	N.A.; AUC (mg·h/L) = 36.88 ± 2.16	43,820 ± 4490	N.A.	[93]
Ursolic acid PEGylated liposome	mice (plasma)	20	intravenous	N.A.; AUC (mg·h/L) = 316.11 ± 3.48	87,150 ± 10480	N.A.	[94]
Ursolic acid FR-targeted liposome	mice (plasma)	20	intravenous	N.A.; AUC (mg·h/L) = 218.32 ± 12.73	109.30 ± 8300	N.A.	[94]

(AUC_0→12_): area under the curve between 0 and 12 h; AUC: area under the curve; C_max_: maximum plasma concentration; T_max_: time to maximum concentration. Values are expressed as the mean ± standard error of the mean when available. N.A.: non-available data.

**Table 6:** Pharmacokinetics parameters of boswellic acids in humans.

Compound	Dose (mg)	Route of Administration	Condition	AUC_0→∞_ (ng·h /mL) (Mean Value)	C_max_ (ng/mL) (Mean Value)	T_max_ (h) (Mean Value)	Reference
Capsule Wok Vel™ (11-keto-β-boswellic acid)	333	oral	N.A.	N.A.; AUC_0→∞_ (µmol/mL·h) = 27.33 × 10^−3^	N.A.; AUC_0→∞_ (µmol/mL) = 2.72 × 10^−3^	4.5	[19]
*B. serrata* dry extract (β-boswellic acid)	786	oral	fasting	6697.1	188.2	4.0	[18]
*B. serrata* dry extract (β-boswellic acid)	786	oral	food	23,316.7	1120.1	8.0	[18]
*B. serrata* dry extract (11-keto-β-boswellic acid)	786	oral	fasting	1660.72	83.8	3.5	[18]
*B. serrata* dry extract (11-keto-β-boswellic acid)	786	oral	food	3037.15	227.1	4.0	[18]
*B. serrata* dry extract (acetyl-11-keto-β-boswellic acid)	786	oral	fasting	153.6	6.0	2.0	[18]
*B. serrata* dry extract (acetyl-11-keto-β-boswellic acid)	786	oral	food	748.9	28.8	3.0	[18]
*B. serrata* dry extract (α-boswellic acid)	786	oral	food	9695	316.7	8.0	[18]
*B. serrata* dry extract (acetyl-α-boswellic acid)	786	oral	food	N.A.; AUC_0→t_ (ng·h /mL) = 1636	118.5	8.0	[18]
Boswelan capsule (11-keto-β-boswellic acid)	800	oral	fasting	859.4	156.7	2.4	[17]
Boswelan capsule (11-keto-β-boswellic acid)	800	oral	food	1179.2	205.7	2.5	[17]
Boswelan capsule (3-acetyl-11-keto-β-boswellic acid)	800	oral	fasting	72.2	30.3	1.9	[17]
Boswelan capsule (3-acetyl-11-keto-β-boswellic acid)	800	oral	food	112.1	32.8	2.1	[17]

(AUC_0→∞_): area under the plasma concentration-time curve from zero to infinity time; (AUC_0→t_): area under the concentration time-curve; C_max_: maximum plasma concentration; T_max_: time to maximum concentration. Values are expressed as the mean ± standard error of the mean when available. N.A.: non-available data.
